# Effect of *Coriandrum sativum* hydroalcoholic extract and its essential oil on acetic acid- induced acute colitis in rats

**Published:** 2016

**Authors:** Bahareh Heidari, Seyed Ebrahim Sajjadi, Mohsen Minaiyan

**Affiliations:** 1 *Isfahan Pharmaceutical Sciences Research Center, School of Pharmacy and Pharmaceutical Sciences, Isfahan University of Medical Sciences, Isfahan, Iran*; 2 *Department of Pharmacognosy, School of Pharmacy and Pharmaceutical Sciences, Isfahan University of Medical Sciences, Isfahan, Iran*; 3 *Department of Pharmacology and Isfahan Pharmaceutical Sciences Research Center, School of Pharmacy and Pharmaceutical Sciences, Isfahan University of Medical Sciences, Isfahan, Iran*

**Keywords:** *Coriandrum sativum*, *Inflammation*, *Colitis*, *Animal model*, *Essential oil*

## Abstract

**Objective:**

The aim of this study was to determine the protective effects of *Coriandrum sativum *on acetic acid-inducedcolitis in rats. *C. sativum* (Coriander) has long been used in Iranian traditional medicine and its use as an anti-inflammatory agent is still common in some herbal formulations.

**Materials and Methods:**

Colitis was induced by intra-rectal administration of 2ml acetic acid 4% in fasted male Wistar rats. Treatment was carried out using three increasing doses of extract (250, 500, 1000 mg/kg) and essential oil (0.25, 0.5, 1 ml/kg) of coriander started 2 h before colitis induction and continued for a five-day period. Colon biopsies were taken for weighting, macroscopic scoring of injured tissue, histopathological examination and measuring myeloperoxidase (MPO) activity.

**Results:**

Colon weight was decreased in the groups treated with extract (500 and 1000 mg/kg) and essential oil (0.5 ml/kg) compared to the control group. Regarding MPO levels, ulcer severity and area as well as the total colitis index, same results indicating meaningful alleviation of colitis was achieved after treatment with oral extract and essential oil.

**Conclusion:**

Since the present experiment was made by oral fractions of coriander thus the resulting effects could be due to both the absorption of the active ingredients and/or the effect of non-absorbable materials on colitis after reaching the colon. In this regard, we propose more toxicological and clinical experiments to warranty its beneficial application in human inflammatory bowel diseases.

## Introduction

*Coriandrum sativum* L. (*C. sativum*) belongs to the family Apiaceae. There are only two known species of the plant* C. sativum* L. (coriander) and its wild relative *C. tordylium*. The fruits (seed and pericarp) are the most widely used components of the coriander plant with the most important constituents being the essential oil and the fatty oil. Coriander, an Italian native plant, is presently cultivated in Central and Eastern Europe, Mediterranean regions, and Asia (Sahib et al., 2012 ▶). Coriander has been known as “Geshniz” in Iran (Asgarpanah and Kazemivash, 2012 ▶). This plant has a high economic value since it is widely used as flavoring agent in food and cosmetics. In Iran, coriander has a long history of medicinal use for preventing convulsions, anxiety, insomnia and loss of appetite. The essential oil content is around 1% and the major component reported in the oil is linalool, in the range of 30–80% of total seed oil (Sahib et al., 2012 ▶). Coriander seeds have a health-supporting reputation. In parts of Europe, coriander has been traditionally referred to as an "anti-diabetic" plant. In the United States, coriander has recently been studied for its cholesterol-lowering effects (Delaquis et al, 2002 ▶).

Coriander, like many spices, contains antioxidants, which can delay or prevent the spoilage of food seasoned with this spice. A study found both the leaves and seed to contain antioxidants (Goswami and Singhai, 2012 ▶). In an early study, administration of coriander seeds to rats fed with a high-fat diet showed decreased levels of peroxides, free fatty acid and glutathione as well as increased activity of antioxidant enzymes (Chithra and Leelamma, 1999 ▶). The radical scavenging activity of coriander oil has been partly attributed to the high composition of un-saponifiables phospholipids present in coriander seed oil. Treatment with polyphenolic fractions of coriander seeds effectively protected human lymphocytes from H_2_O_2_-induced oxidative stress and restored oxidative status to that of normal cells (Hashim et al., 2005 ▶). According to evidence coriander possesses hepatoprotective activity against carbon tetrachloride (CCL4) intoxication, *in vivo* (Pandey et al., 2011 ▶).

The use of coriander as an anti-inflammatory agent is evident by a traditional formulation from Sri Lanka, Maharasnadhi Quather (MRQ), containing coriander seeds as one of its principal components. MRQ has been reported to have analgesic and anti-inflammatory properties both in animal models and human subjects. Administration of MRQ significantly inhibited carrageenan-induced rat paw edema. The formulation also increases pain tolerance in rats by 57% after 1 h of treatment as assessed by the hot plate test (Thabrew et al., 2003 ▶).

Inflammatory bowel disease (IBD) is a widely chronic and multifactorial gastrointestinal (GI) inflammatory condition which is categorized into ulcerative colitis and Crohn's disease in the clinic. Etiology and pathophysiology of IBD is still unknown and multifactorial (Sellin and Pasricha, 2006 ▶). Intestinal mucosal inflammation as a characteristic feature of IBD is induced by an increase in the activity of some mucosal immune cells where the T-helper cells play an important role (Sartor RB, 1997 ▶) (Bouma and Strober, 2003 ▶).

Sulfasalazine, mesalamine and 5-ASA derivatives, glucocorticoides and immune- suppressive agents are among the current medications for which limited efficacy and various side effects are commonly reported (George and Chrousos, 2009 ▶). Because of lack of specific and curative treatments with low toxicity, there is a growing need to develop safe and effective treatments for IBD (Sellin and Pasricha, 2006 ▶). The present study on the inhibition of ulcerative colitis damage by coriander was undertaken considering the following points: (1) The widespread presence of coriander in frequently consumed foods, pharmaceutical preparations and cosmetics., (2) The use of coriander in folk medicine as an aromatic carminative, stomachic, anti-inflammatory and antispasmodic and a remedy against gastrointestinal discomforts such as dyspepsia and flatulence; and (3) The antioxidant nature of its constituents (Al-Mofleh et al., 2006 ▶). The aim of this study was to evaluate protective and therapeutic effects of essential oil and hydroalcoholic extract of the fruits of *C. sativum* via oral administration in an acetic acid model of colitis.

## Materials and Methods

**Chemicals**

Prednisolone powder was prepared as gift from Iran Hormone Pharmaceutical Co. (Tehran, Iran). Hexadecyltrimethyl ammonium bromide, O-dianisidine dihydro-chloride as well as organic solvents and acetic acid were purchased from Merck Company (Darmstadt, Germany).

**Plant material and preparation of extract and essential oil**

Coriander fruits were purchased from Pakanbazr Co. (Isfahan, Iran) in May 2013. The plant identity was confirmed by Pharmacognosy Department of School of Pharmacy, Isfahan University of Medical Sciences, Isfahan, Iran. Herbarium voucher No. 2082 was deposited in the Pharmacognosy department.

For preparation of hydroalcoholic extract, coriander fruit powder (400g) were macerated with 2160 ml of EtoH-H20 (80:20) for 24 hours. The extract was then shaken, filtered and evaporated in a rotary evaporator under reduced pressure until a semisolid extract was obtained. Moreover, the concentrated extract was freeze-dried to obtain a dry powdered extract with yield value of 10/85 % (W/W). Essential oil of coriander was isolated by hydrodistillation of the fruits powdered of the plant using a clevenger type apparatus during 3 h in a full glass apparatus, according to the method reported by Ghannadi (Ghannadi et al., 2002 ▶; Iranian Herbal Pharmacopoeia, 2002 ▶).

**Animals**

Fifty four male Wistar rats (225± 25g body weight) obtained from animal house of Isfahan School of Pharmacy (Isfahan, Iran) were allowed to adapt to the laboratory environment for one week. They had free access to tap water and rat chow pellets and were housed in plexy glass cages under controlled conditions of temperature (20-22 °C), humidity and light/dark (12/12 h) cycles. The experiments were done according to the guidelines provided by Ethics and Research Committee of Isfahan University of Medical Sciences, Isfahan, Iran. 

**Animal grouping**

Nine groups of rats with 6 animals in each were studied. Normal group: Normal rats without ulcer induction received vehicle (2ml/kg normal saline, p.o.). Control colitis group: Rats with induced colitis received vehicle (2ml/kg, p.o). Reference group: Rats with induced colitis received prednisolone (4mg/kg, p.o.). Test groups: Rats with induced colitis received increasing doses of *C. sativum* hydroalcoholic extract (CSHE) (250, 500, 1000 mg/kg, p.o.). Rats with induced colitis received increasing doses of *C. sativum* essential oil (CSEO) (0.25,0.5, 1 ml/kg, p.o.). All the treatments were carried out 2 hours before colitis induction and continued for 4 days on a daily basis. Finally, the animals were euthanized by ether overdose inhalation 24 hours after the last dose (a period of five-day treatment).

**Experimental protocol**

Test samples including solutions or suspensions of drug or plant extract were freshly prepared. The plant extract was prepared as a suspension in 0.2% V/V Tween 80. Acute colitis was induced by 2 ml acetic acid (4%) using a technique which was first introduced by Mascolo et al. (Mascolo et al. 1995 ▶). Briefly, the rats were fasted for 24 hours with free access to tap water and observed to ensure their health before induction of colitis. The rats were lightly anesthetized with ether. A flexible plastic rubber catheter with an outside diameter of 2mm was inserted 8cm into the colon and then the animals were maintained in a head-down position for 2 min to prevent solution leakage. In Sham operated and control groups, normal saline (2ml/kg) was instilled. Colon biopsies were taken for macroscopic scoring of injured tissue, histopathological examination and measuring myeloperoxidase (MPO) activity.

**Assessment of colon macroscopic damage**

The abdomen was opened and the colon, 8cm in length and 2cm proximal to the anus, was excised and incised longitudinally and washed with normal saline. Wet colon was then weighed and its changes were determined for each group. Photos of colon segments were taken by a Sony camera, transferred to a personal computer and analyzed subsequently by Fiji Image Processor Program for measuring the ulcerated areas (Minaiyan et al., 2006 ▶). Then, macroscopic mucosal damage was evaluated using a validated grading scale according to Morris et al. (Morris et al., 1989 ▶). Scores were: 0=no ulcer, 1=mucosal edema, 2=slight bleeding or erosions, 3=moderate edema, bleeding ulcers or erosions, 4=severe ulceration, erosions, edema and tissue necrosis or perforation.

Ulcer index was determined by summing the ulcer score and the ulcer area for each colon. For further assessments, tissue samples were cut into two equal parts longitudinally, a part was stored immediately at -20 °C till biochemical analysis (MPO determination) and the other parts were stored in 10% formalin for pathological evaluation (Motavallian Naeini et al., 2012 ▶).

**Assessment of colon pathology**

Fixed colon tissue was dehydrated; paraffin embedded, processed, sectioned in 4 µm thick samples, and stained with haemotoxylin and eosin (H&E). Inflammation and crypt damage were assessed on H&E-stained and coded sections using a validated scoring scheme set up in our laboratory as described by Cooper et al. and Dieleman et al. (Cooper et al.1993 ▶; Dieleman et al. 1998 ▶). Total colitis score as the sum of the 3 following sub-scores (inflammation severity, inflammation extent, and crypt damage) was finally measured for each specimen. Pathological evaluation and scoring was performed using a Zeiss microscope equipped with a Sony color video camera for digital imaging.

**Assessment of colonic MPO activity**

MPO activity, a marker of polymorph-nuclear leukocyte migration, was determined using a previously described method (Morris et al., 1989 ▶). To measure the enzymatic activity of MPO, samples were removed from the freezer and chopped into small pieces after melting. Then, a total amount of one hundred milligrams of colon mucosal scraping was homogenized in a solution filled 0.5% hexadecyltrimethyl ammonium bromide dissolved in 50 mM potassium phosphate buffer (pH=6), before sonication in an ice bath for 45s for four times. The homogenates were freeze-thawed for three times. Then, sonication was repeated and samples were centrifuged for 15 min at 15000 rpm.

The level of MPO activity was measured at 450 nm by spectrophotometer (UNICO- spectrophotometer, 2100 UV/VIS).For this purpose, 0.1 ml of the solution was mixed with 2.9 ml of 50 mM phosphate buffer, pH 6.0, containing 0.167 mg/ml O-dianisidine dihydrochloride. 

MPO activity was defined as the quantity of enzyme degrading 1 mM of peroxide per minute at 25 C and was expressed in units per gram (U/g) of wet tissue.

**Statistical analysis**

Statistical analysis was performed using SPSS 14.0 statistical software. Differences among groups were compared using parametric one-way analysis of variance (ANOVA) with Tukey HSD as post hoc test. Non-parametric data was analyzed by Mann-Whitney U test. Results are shown as the mean ± SEM. The significance was identified at P<0.05.

## Results

**Analysis of** ***C. sativum*** **essential oil**

Major constituents of the essential oil were shown in Table 1. As it is shown, linalool,  - terpinene and α- pinene are the three most abundant compounds in corindrum essential oil. Geranyl acetate, p-cymene and β- pinene are other constituents that could be found in coriander

**Colon weight variation**

The results showed that colon weight was decreased in the groups treated with the extract at the doses of 500 mg/kg (P<0.05) and 1000 mg/kg (P<0.001) compared to untreated control group (Table 2). In essential oil-treated groups, same result was found at the dose of 0.5 ml/kg (p<0.05) while larger and smaller doses were not effective (p>0.05). Prednisolone-treated group demonstrated significant decrease in colon weight (p<0.01) in comparison to control group (Table 2).

**Table 1:** Percentage composition of the essential oil of *C. sativum *(coriander

**No.**		**Compound**	**%**	**RI**		**No.**		**Compound**	**%**	**RI**
**1**		Hexanal	t	801		21		Borneol	0.2	1170
**2**		Heptanal	t	902		22		4-terpineol	0.5	1180
**3**		α-thujene	0.3	930		23		α-terpineol	0.3	1192
**4**		α-pinene	8.8	940		24		Decanal	0.4	1205
**5**		Camphene	0.3	952		25		Citronellol	0.5	1229
**6**		Benzaldehyde	t	962		26		Geraniol	1.2	1256
**7**		Sabinene	0.9	976		27		2-*E*-decenal	0.3	1262
**8**		β-pinene	1.9	979		28		n-decanol	t	1272
**9**		Myrcene	0.8	991		29		Undecanal	t	1304
**10**		Octanal	t	1001		30		citronellyl acetate	0.1	1353
**11**		α-terpinene	0.2	1018		31		neryl acetate	t	1364
**12**		p-cymene	3.7	1027		32		Geranyl acetate	5.4	1383
**13**		Limonene	0.7	1031		33		Dodecanal	0.2	1406
**14**		*trans*-β-ocimene	t	1049		34		β-caryophyllene	0.2	1416
**15**		γ-terpinene	10.2	1062		35		2-E-dodecenal	1.6	1465
**16**		*trans*-linalool oxide	0.2	1074		36		Methyl tetradecanoate	t	1720
**17**		Terpinolene	0.1	1088		37		Tetradecanoic acid	0.5	1765
**18**		Linalool	59.2	1098		38		methyl hexadecanoate	t	1924
**19**		Camphor	0.5	1149		39		Hexadecanoic acid	0.2	1969
**20**		Citronellal	0.4	1156						

RT: Retention time, RI= Retention index on HP-5MS capillary column, t=trace (<0.05%), Percentages calculated from TIC data.

**Table 2:** Effects of *C. sativum* hydro-alcoholic extract (CSHE) and essential oil (CSEO) on macroscopic parameters of colitis induced by acetic acid in rats

**Groups**	**Score ** **(0-4)**	**Ulcer Area ** **(Cm** ^2^ **)**	**Total Colitis Index** **(0-12)**	**Colon weight** **(Mg)**	**Groups**	**Score ** **(0-4)**	**Ulcer Area ** **(Cm** ^2^ **)**	**Total Colitis Index** **(0-12)**	**Colon weight** **(Mg)**
**Normal**	0.0±0.0	0.0±0.0	0.0±0.0	0.0±0.0	CSHE 1000	1.1±0.3**	2.9±0.9***	4.0±1.1***	110±0.2***
**Colitis**	4.0±0.1	8.0±0.3	12.0±0.4	190±0.1	CSEO 0.25	2.2±1.3	6.3±1.1	8.5±1.3	160±0.1
**Pred.4**	1.7±2.1*	4.4±0.6*	6.1±0.6*	110±0.1**	CSEO 0.5	0.8±1.1**	3.4±1.2**	4.2±1.5***	130±0.1*
**CSHE 250**	2.2±1.1	5.2±0.6	7.4±0.5	160±0.1	CSEO 1	1.0±0.8**	4.8±0.7	5.8±0.7**	140±0.1
**CSHE 500**	0.6±0.5**	1.4±0.5***	2.0±0.7***	130±0.1*					

Data are expressed as Mean ± SEM., P.O. = oral, Pred. =Prednisolone (4mg/kg). CSHE (250, 500, 1000 mg/kg), CSEO (0.25, 0.5, 1 ml/kg) (n=6).

*: p<0.05,

** : p<0.01,

*** : p<0.001 denote significant difference versus control group.

**Macroscopic and histopathology presentation**

**Figure 1:**
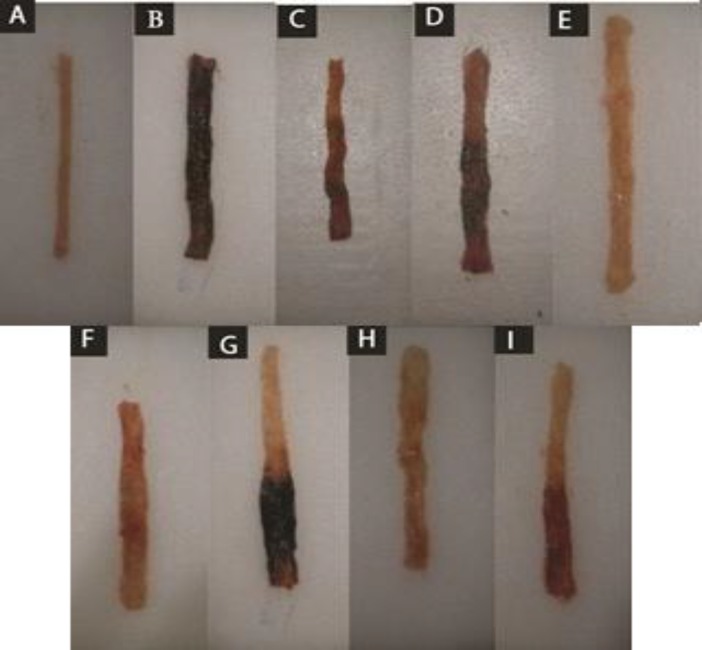
Macroscopic presentation of acetic acid-induced colitis in rats. A: Normal colon treated with normal saline (2 ml/kg). B: Control colitis treated with normal saline (2 ml/kg). C: Prednisolon treated colitis (4 mg/kg). D: *C. sativum* hydro-alcoholic extract-treated colitis (250 mg/kg) E: *C. sativum* hydro-alcoholic extract -treated colitis (500 mg/kg) F: *C. sativum* hydro-alcoholic extract-treated colitis (1000 mg/kg) G: *C. sativum* essential oil-treated colitis (0.25 ml/kg) H: *C. sativum* essential oil -treated colitis (0.5 ml/kg) I: *C. sativum* essential oil-treated colitis (1.0 ml/kg

**Figure 2:**
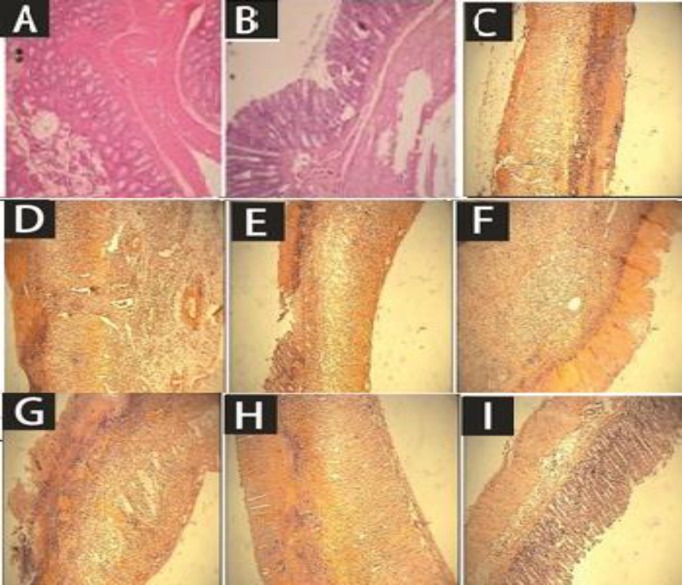
Microscopic presentation of acetic acid-induced colitis in rats (H&E staining with 40x magnification). A: Sham, normal colon treated with normal saline, 2 ml/kg; mucus layer and crypts are normal and leukocyte infiltration is absent. B: Control colitis treated with normal saline, 2 ml/kg; mucosal and sub-mucosal inflammation as well as crypt damage and leukocyte infiltration are completely evident; C: Prednisolone-treated colitis, 4 mg/kg. D: Colitis treated with *C. sativum* hydro-alcoholic extract (CSHE), 250mg/kg, p.o.; E: 500 mg /kg, p.o.; F: 1000 mg/kg administered p.o. G: Colitis treated with *C. sativum* essential oil (CSEO), 0.25 ml/kg, p.o.; H: 0.5 ml/kg, p.o.; I: 1 ml/kg, p.o

Similar to above-mentioned results, macroscopic parameters including ulcer severity (US), ulcer area (UA), and ulcer index as well as the total colitis index were improved in groups treated with two greater doses (500, 1000 mg/kg) of coriander extract and middle dose of essential oil (0.5 mg/kg) (Tables 2 and 3). Essential oil of coriander (1.0 ml/kg) was also effective to alleviate ulcer severity and total colitis index (p<0.01) (Tables 2 and 3). Prednisolone, as the reference drug was able to improve most of macroscopic and microscopic parameters as it is shown in Figures 1 and 2.

**Table 3:** Effects of *C. sativum* hydro-alcoholic extract (CSHE) and essential oil (CSEO) on total colitis index of rats.

**Groups**		**Total colitis** ** Index (0-10)**
**Normal**			0.0±0.0
**Colitis**			8.3±0.0
**Pred.4**			3.5±0.4**
**CSHE 250**			3.7±0.3**
**CSHE 500**			3.8±0.3**
**CSHE 1000**			3.5±0.2**
**CSEO 0.25**			5.4±0.4
**CSEO 0.5**			3.7±0.3**
**CSEO 1**			3.8±0.3**

Data are expressed as mean ± SEM. Pred.= Prednisolone (4mg/ kg), CSHE (250, 500,1000 mg/kg) CSEO (0.25, 0.5, 1.0 ml/kg)(n=6). All treatments were done orally.

** p<0.01 denotes significant difference versus control group.

**Biochemistry assessment**

**MPO levels**

**Figure 3:**
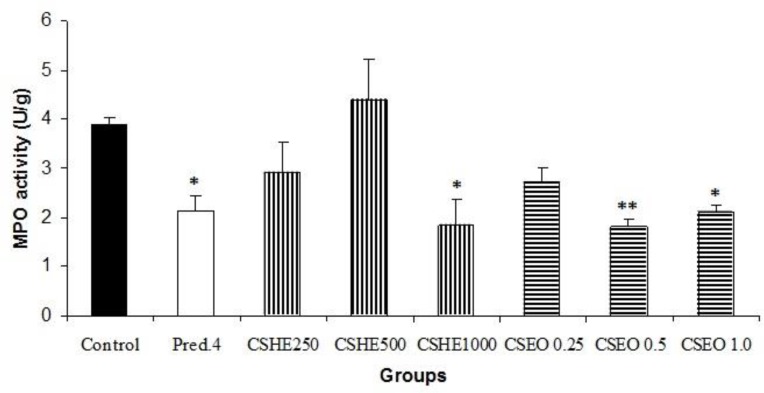
Myeloperoxidase (MPO) activity in groups treated with increasing doses of oral *C. sativum* extract (CSHE, 250, 500, 1000 mg/kg), essential oil (CSE, 0.25, 0.5, 1 ml/kg) and prednisolone (4mg/kg). Data are presented as mean ± SEM. *p<0.05 and **p<0.01 denote significant differences compared to control group

In the group treated with the extract (1000 mg/kg), compared to the control group, a significant (p<0.05) decline in MPO level is evident. In this regard, the essential oil at the doses of 0.5 and 1 ml/kg is also able to reduce MPO activity. Prednisolone-treated group experienced a significant effect (p<0.05) in this regard (Figure 3).

## Discussion

In the present study, method of acetic acid that is both rapid and reproducible was used for inducing diffuse colonic inflammation which resembles many histological characteristics of human ulcerative colitis (McPherson and Pfeiffer, 1978 ▶). 

Results of our study showed that the doses of 500 and 1000 mg of coriander extract and 0.5 and 1 ml of the essential oil were thoroughly effective on evaluated lesions of colitis. Some of these parameters are directly related to inflammatory basis of colitis e.g. ulcer index and total colitis index while the others may indirectly assess the inflammatory nature of colitis and oxidative stress magnitude, like colonic weight and MPO activity, respectively (Motavallian-Naeini et al., 2012 ▶ ). Therefore, it is suggested that both coriander extract and essential oil ingredients are bioavailable and biologically active after oral intake. Among most of evaluated parameters, the best result was obtained with the middle dose of coriander extract or essential oil *i.e.* doubling the dose did not cause two-fold increases in protective response against tissue. However, in the case of the lowest dose, the results indicated that the dose had an important role. Indeed, for most of the evaluated parameters, the lowest dose of coriander extract and volatile oil were not effective on colitis parameters (Tables 2-4), although the differences between the two higher doses were not statistically significant. This may suggest the presence of some active materials in examined fractions for which opposite activity will be accentuated at greater doses (Minaiyan et al., 2014 ▶). It seems that more doses should be tried to demonstrate the exact dose-effect relationship of coriander in further studies. 

Coriander has been traditionally used to treat inflammatory diseases like gout and rheumatism because of its anti-inflammatory properties (Varier PS, 1994 ▶) so its seeds has been listed in the European Pharmacopoeias and used as a digestive aid to treat rheumatism (Al Rowais NA, 2002 ▶).

Linalool and linalyl acetate are the main components of coriander essential oil known to possess several biological activities e.g. anti-oxidant, anti-microbial, hypoglycemic, hypolipidemic, anxiolytic, analgesic and anti-inflammatory effects (Laribi et al., 2015 ▶; Sahib et al., 2013 ▶). Linalool, on the other hand is the most abundant constituent (in this study, approximately 60%) that may reach to 70% of total coriander essential oil in some cases (Burdock and Carabin, 2009 ▶). So, it is plausible to accept that linalool plays a principal role in bioactivity represented by coriander essential oil. Much lesser percentages were reported for  γ-terpinene (10.2% ), α-pinene (8.8% ) and geranyl acetate (5.4%), which almost resemble the data reported in a previous study (Asgharpanah and Kazemivash., 2012 ▶).

Peana et al. reported that linalool plays a major role in the anti-inflammatory activity displayed by the herbal essential oils, and provided further evidences suggesting that linalool and linalyl acetate-producing herbal species are potential anti-inflammatory agents (Peana et al., 2006 ▶). Furthermore, safety assessment of coriander essential oil as a food ingredient has been done by Burdock and Carabin. They demonstrated that coriander oil is neither a sensitizer nor a toxic agent (up to 500 mg/kg/d) at conventional doses so it could be used as a food additive (Burdock and Carabin, 2009 ▶). 

Hydroalcoholic extract of *C. sativum* (CSHE) was the other fraction assessed in our study. We know that this fraction is a potential source of lipids like linoleic acid and petroselinic acid isolated from the seeds and the aerial parts of the plant (Sahib et al., 2013 ▶; Laribi et al., 2015 ▶). Due to the presence of a multitude of bioactive materials, several pharmacological effects have been ascribed to coriander total extract which for most cases, are similar to those mentioned for essential oil (Hwang et al., 2014 ▶; Goswami and Singhai, 2012 ▶). For instance, in a study by Nair et al., the anti-inflammatory activity of CSHE was evaluated using carrageenan-induced paw edema model and the anti-granuloma activity of that was evaluated by determining serum tumor necrosis factor (TNF)-α, interleukin (IL)-6, IL-1 β levels, as markers of global inflammation. *C. sativum *hydroalcoholic extract produced a significant reduction (P < 0.05) in paw edema after carrageenan administration (Nair et al., 2013 ▶).

The key role of cytokines such as TNF-α, IL-6, IL-1 β and leukotriens in the pathogenesis of IBD, particularly ulcerative colitis suggests that coriander may act through decreasing the synthesis or function of cytokines. We know that glucocorticoides are among powerful anti-inflammatory drugs that have beneficial effects in sever IBD (Kenneth and McQuaid, 2009). Since glucocorticoids are powerful inhibitors of cytokines synthesis and/or activity, prednisolon was applied in our study as the reference drug (George and Chrousos, 2009 ▶). In favor of this hypothesis the results of Wu et al. showed that coriande has a strong anti-inflammatory property as it inhibits pro-inflammatory mediator expression by suppressing necrosis factor (NF)-kappa B activation in lipo-poly saccharides (LPS)-induced macrophages. In their study, ethanol extracts from both stem and leaves of *C. sativum* significantly decreased LPS-induced nitric oxide and prostaglandin E-2 production as well as inducible nitric oxide synthesis (Wu TT et al., 2010 ▶). Considering the role of NF-kappa B, LPS, PGE-2 and nitric oxide (NO) in the pathogenesis of IBD and inhibitory effects of coriander active components on them, beneficial results from examined fractions in current study could be warranted. 

The result of our study validated traditional use of coriander for the management of inflammatory bowel disorders and demonstrated the anti-inflammatory and anti-colitis activities of coriander in an experimental model of acute colitis. Therefore, multiple pharmacological effects  of coriander including  anti-inflammatory, analgesic,  anti-oxidant, and antispasmodic as well as its wide array of uses especially as an edible vegetable or food spice suggests it as a good candidate for IBD prevention or therapy in human (Meixia et al., 2013).
